# A gender- based approach to the current situation of Spanish dentists

**DOI:** 10.4317/jced.58303

**Published:** 2021-09-01

**Authors:** Rocío E. Hernández-Ruiz, Cristina Benavides-Reyes, Santiago González-López, Mª Victoria Bolaños-Carmona

**Affiliations:** 1 Department of Operative Dentistry. School of Dentistry. University of Granada, Campus de Cartuja. Colegio Maximo s/n, E-18071, Granada, Spain; 2 Department of Pediatric Dentistry, School of Dentistry, University of Granada, Campus de Cartuja. Colegio Maximo s/n, E-18071, Spain

## Abstract

**Background:**

To determine the perception of Spanish dentists about the situation of the profession nowadays and how the changes occurred in dental workforce (in number and gender of the past twenty years) have affected their personal and professional lives, under a gender-based approach.

**Material and Methods:**

An online survey comprising of 51 opinion and socio-economic questions, divided into 9 sections of different topics, which was administered between the members of 13 professional associations. Chi-squared tests were calculated (*p*<0.05).

**Results:**

Valid responses were received from 422 participants with a mean age of 41 years old and 66% of female dentists. Most dentists considered their selves in a “good” position, however, 72.3% of them said the profession “has had worsened”. Opinions significantly differed between women and men in gender equity and the pay gap between them could be observed (29.4% of males earned more of 4000 euros a month, while only 15.1% female dentists did). Also, 49.5% of female dentists felt underrepresented in the highest association of Spanish dentists (General Council of Dentist - Consejo General de Dentistas) and 38.4% declared they have had suffered verbal violence coming from patients repeatedly.

**Conclusions:**

A generally negative perception of dentists’ work quality was found among respondents. Also, opinions between males and females differed in important aspects of professional development. Further research projects are needed to have growing evidence on problems and disparities in the dental workforce which would help the institutions to make improvement actions.

** Key words:**Dentists, Health Workforce, Survey, Gender, Spain.

## Introduction

Nowadays, health workforce is mainly feminine, with around 67% of health professionals being women. However, there are still gender inequalities based on the country or the job, among other reasons. Women are being in general, overrepresented in the less qualified healthcare professions and underrepresented in the positions of higher incomes and responsibilities (as doctors, pharmacists or dentists) (1).

In Spain in 2018, there was a feminine majority in all health professions (except for veterinarians, dental technicians and physics with medical specialty). Between dentists, women addressed 56.3% of the practitioners and they were more than men in all ranges under 55 years old (2).

Therefore, the country is one of the global exceptions as the Scandinavian countries or the United Kingdom are, with more women than men practicing medicine and dentistry, due to the increasing number of women entering in medical schools during last years (3,4).

However, in these countries as in less developed ones, there are still professional practices that perpetuate gender inequities between healthcare workers, as the so-called “glass ceiling effect” (5). It can be observed that women have more part-time jobs than men, they specialized less in their fields and they are fewer in leadership and high-ranking positions. These trends enlarge the pay gap in the healthcare sector, in which with the same working hours and position, it is estimated that women earn 11% less than their men counterparts (being up to 28% less in general) (1).

The importance for the healthcare system (and therefore the dental sector) of getting rid of these inequities lies in better working conditions for women will bring an increase in productivity, distribution, motivation and retention of workers (6).

Likewise, addressing gender trends in health workforce is needed to progress towards universal health coverage and the Sustainable Development Goals (SDGs) for 2030 adopted for Spain and 192 countries in the world (7,8). It’s particularly related with the achievement of SDG 3 (health and wellbeing), 5 (gender equity), 8 (decent work and economic growth) and specifically with the objective 3.c that refers to improve development, training, recruitment and retention of the health workers (6).

Gender and their trends are always dynamic and they are linked to societies and their characteristics (9). Therefore, possible gender inequities existing between healthcare providers need to be analysed and understood inside of their particular context, like the type of healthcare system, political regime, economy or culture of the region (3,10).

To obtain organised data of health workforce characteristics is needed to implement effective politics, based on evidence, which would be able to improve citizens’ health coverage, which would generate improvements in social, economic and health environments (11,12). Available information about dentists in Spain is very limited, reduced to global numbers and percentages of dentists per region, sex and age ranges (13,14).

On the other hand, the way the dental sector is orgfanised in the country, based in small dental private practices and every day more often in chain practices owned by corporations, and the lack of sanitary regulation from the health and professional authorities in the field, have generated that the organization and distribution of dentists rely only on the market forces (15). This has resulted in uncontrolled proliferation of dentists, being the health profession of the country with the biggest variation of quantity of professionals (15), that have doubled in the last twenty years, as the General Council of Spanish Dentists predicted in 2010 (16). This process of “commercialising” in which dental professionals are involved, could be having negative consequences to their labour, social or economic conditions. Among others, earnings, life quality or equal opportunities could being affected.

The objective of this research is to know the perception of Spanish dentists about their professional and personal situation, from a gender perspective.

## Material and Methods

A cross-sectional study was carried out collecting data from registered Spanish dentists that were members of a professional college, which were invited by email to fill in an online survey. Our study accomplishes to Helsinki Declaration and we follow STROBE guidelines for cross-sectional studies (17) and non-sexist use of language guidelines.

- Study population and sample.

The population of interest was the total of professionally registered dentists in Spain (being part of a professional association is a legal requirement to work as a dentist) that were 38.999 people in May 2020 (18). All professional associations were contacted except two of them that were excluded (because they had less than 50 dentists). Thirteen associations were favourable to the survey distribution between their dentists but the way of distribution varied, nine of them delivered the survey in an individual email and four inside of a newsletter of the month. The final sample number was 6375 people invited to participate directly and 2778 indirectly.

- Survey design.

A search on previous similar questionnaires was carried out and it was decided to adapt the one conducted by Metroscopia in 2017 for the General Council of Spanish Lawyers (19) to the dental profession characteristics. Its design was also adapted to the online platform for surveys called Limesurvey (LimeSurvey GmbH, Hamburg, Germany. http://www.limesurvey.org).

Finally, a pilot test was carried out in a group of twelve dentists that were actively working in February of 2020 (ages ranged from 23 to 60 years old). After this pre-test, some adjustments were made based on the feedback of the participants and the final version of the questionnaire was obtained (Table 1,  Table 1 cont.,  Table 1 cont-1.,  Table 1 cont-2.). It was composed of 51 questions, grouped by similar topics in 9 sections: 1) Basic data of the participant, 2) Current situation of dentistry in Spain, 3) Factors for professional success, 4) Equal opportunities in dentistry, 5) Balancing work and family life, 6) Actions to promote gender equity in dental institutions, 7) Professional situation, 8) Socio-demographic data and 9) Gender discrimination.

**Table 1:**
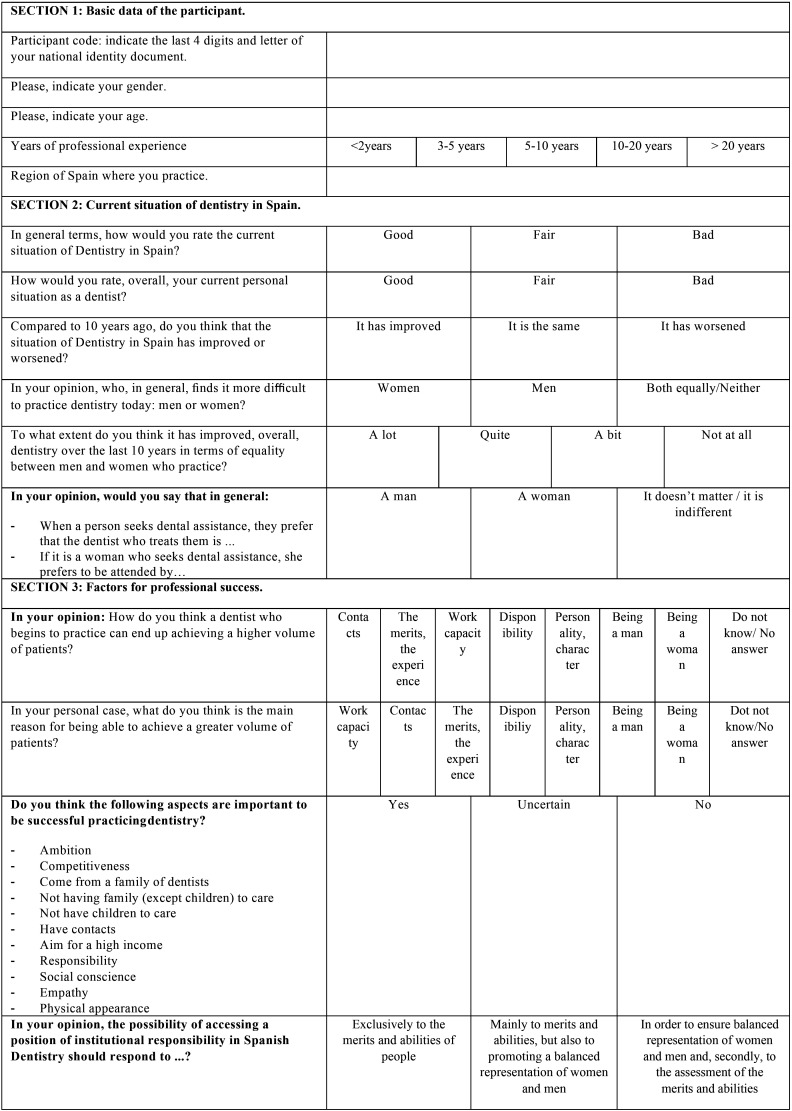
Questionnaire distributed among Spanish dentists. The questions were adapted for the authors from the study of Camas-García *et al*. in 2015.

**Table 1 cont.**
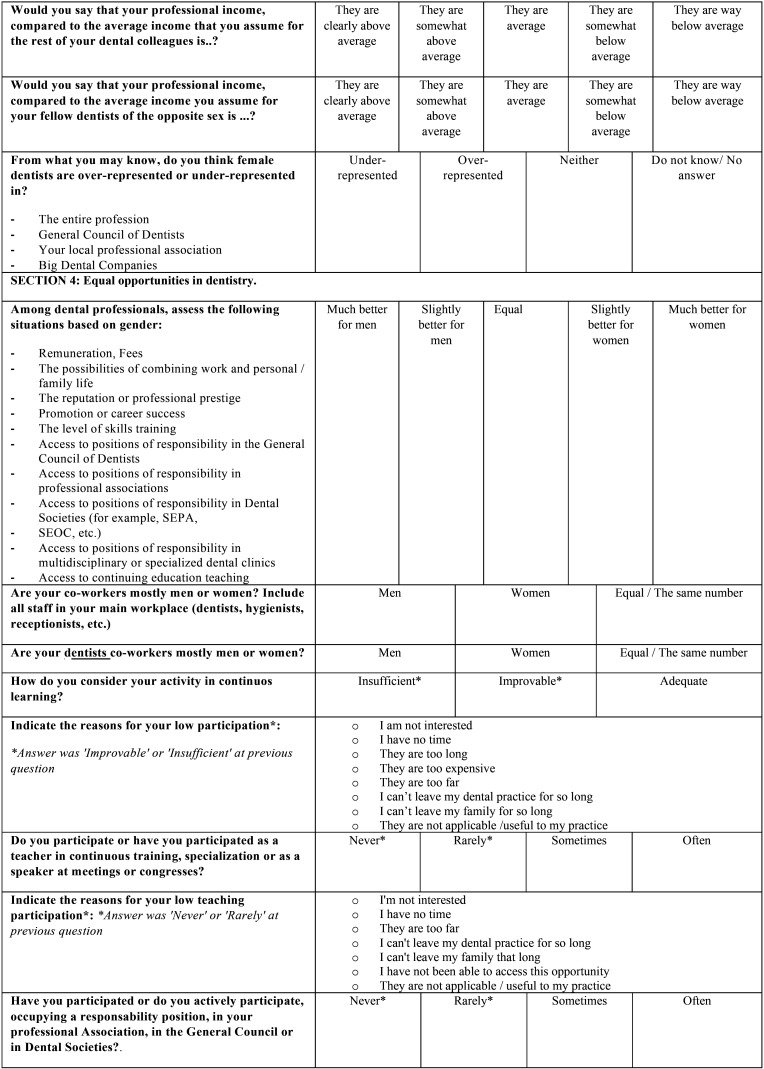
Questionnaire distributed among Spanish dentists. The questions were adapted for the authors from the study of Camas-García *et al*. in 2015.

**Table 1 cont-1.**
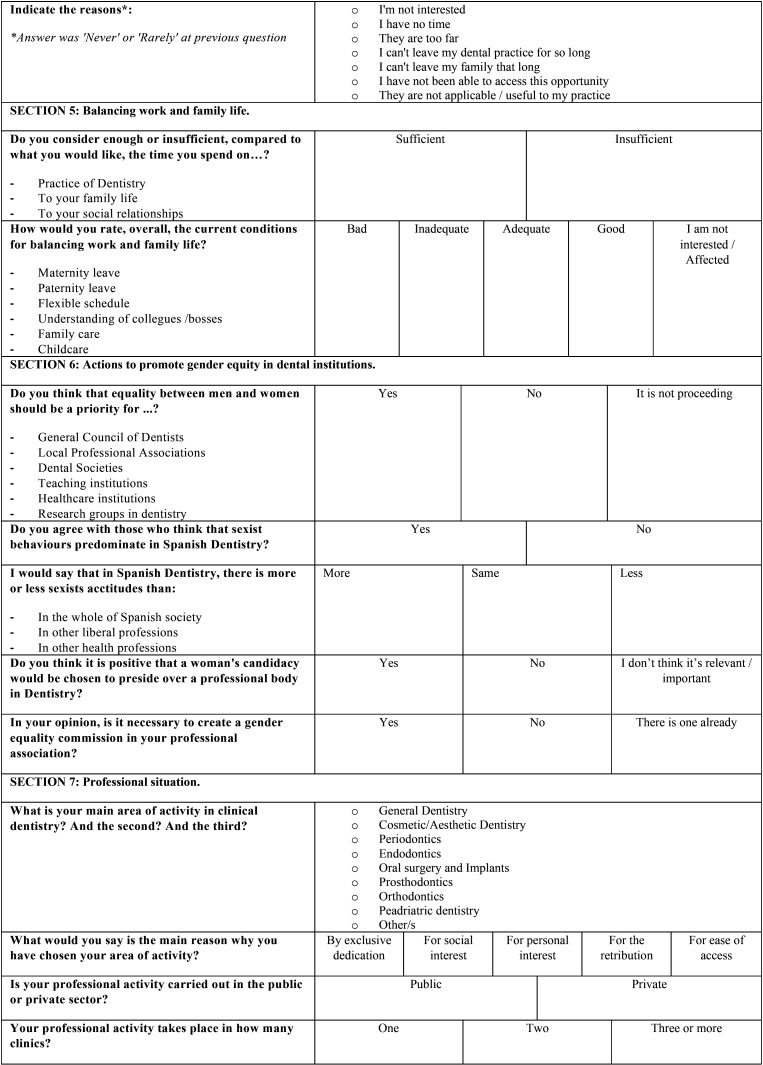
Questionnaire distributed among Spanish dentists. The questions were adapted for the authors from the study of Camas-García *et al*. in 2015.

**Table 1 cont-2.**
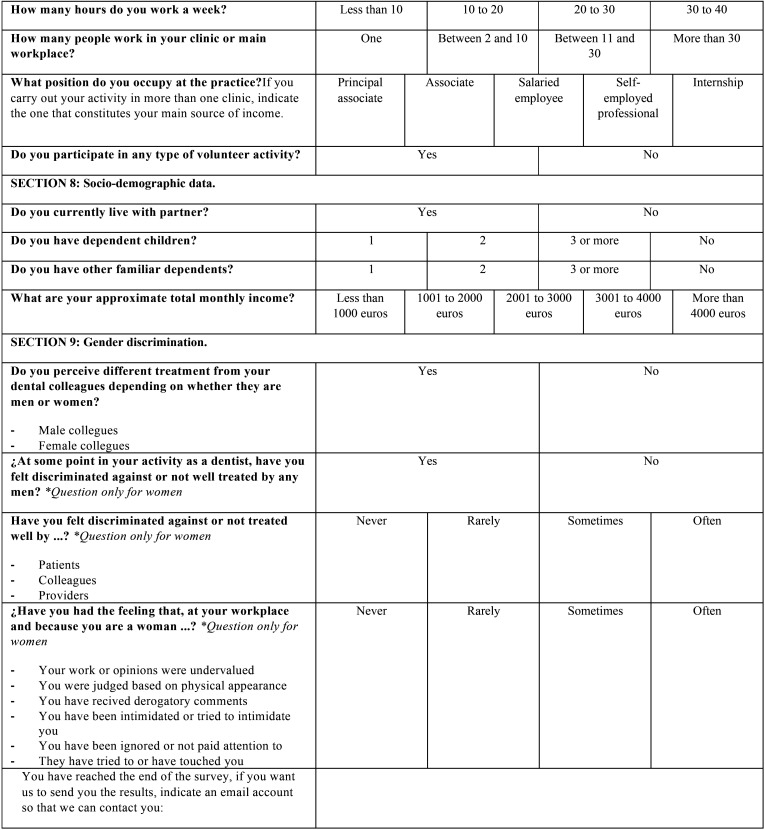
Questionnaire distributed among Spanish dentists. The questions were adapted for the authors from the study of Camas-García *et al*. in 2015.

Dentists invited to participate were informed about the characteristics of the survey before accessing the questions: approximate time required, anonymous responses, researchers’ information and treatment of personal data. Informed consent was obtained from all participants before collecting their responses. Responses were recorded from February to April of 2020.

- Statistical analysis.

Data treatment and analysis were performed with SPSS version 21.0 (IBM-SPSS Inc., Chicago, USA). A descriptive analysis was carried out comparing the frequencies of the obtained responses and the gender using Chi-squared test. Tests to assess the internal consistency (Cronbach’s alfa) and validity (factorial analysis tests) were conducted to the questionnaire. Statistical significance was established with *p* value <0.05.

## Results

From 720 responses, 422 were valid and 338 were excluded because they were uncompleted. Cronbach’s alfa coefficient resulted in satisfactory internal consistency (0.813). Validation tests showed a total explicated variance of 68,44% (using Varimax rotation), an acceptable Kaiser-Meyer-Olkin measure (0.773) and a statistically significative Bartlett’s test (<0.001).

Principal socio-demographic characteristics of respondents were analysed and are presented in Table 2. Age average of participants was 41 years old, observing statistically significant differences between genders (Z=-4.548; *P*<0.001), 50% of women that answered had 37 years old or less while men had 46 years old or less.

**Table 2:**
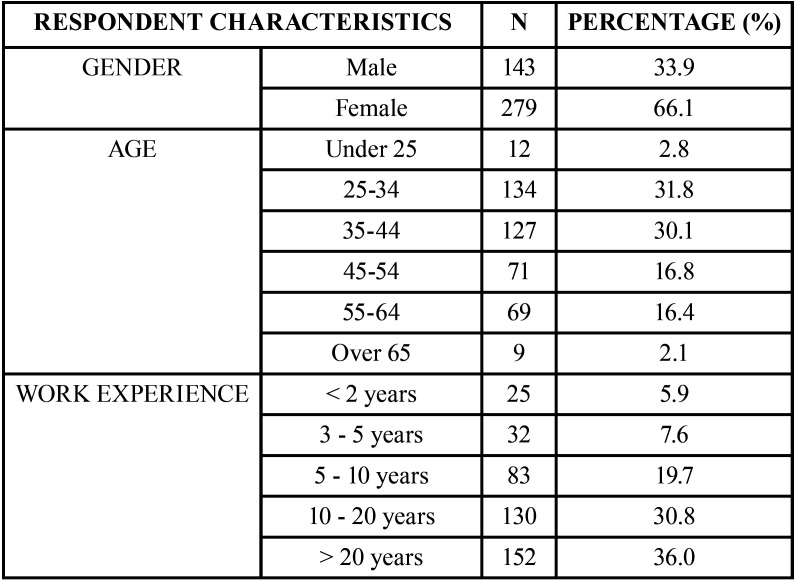
Summary of main characteristics of respondents.

- Current situation of Dentistry in Spain.

**Figure 1:**
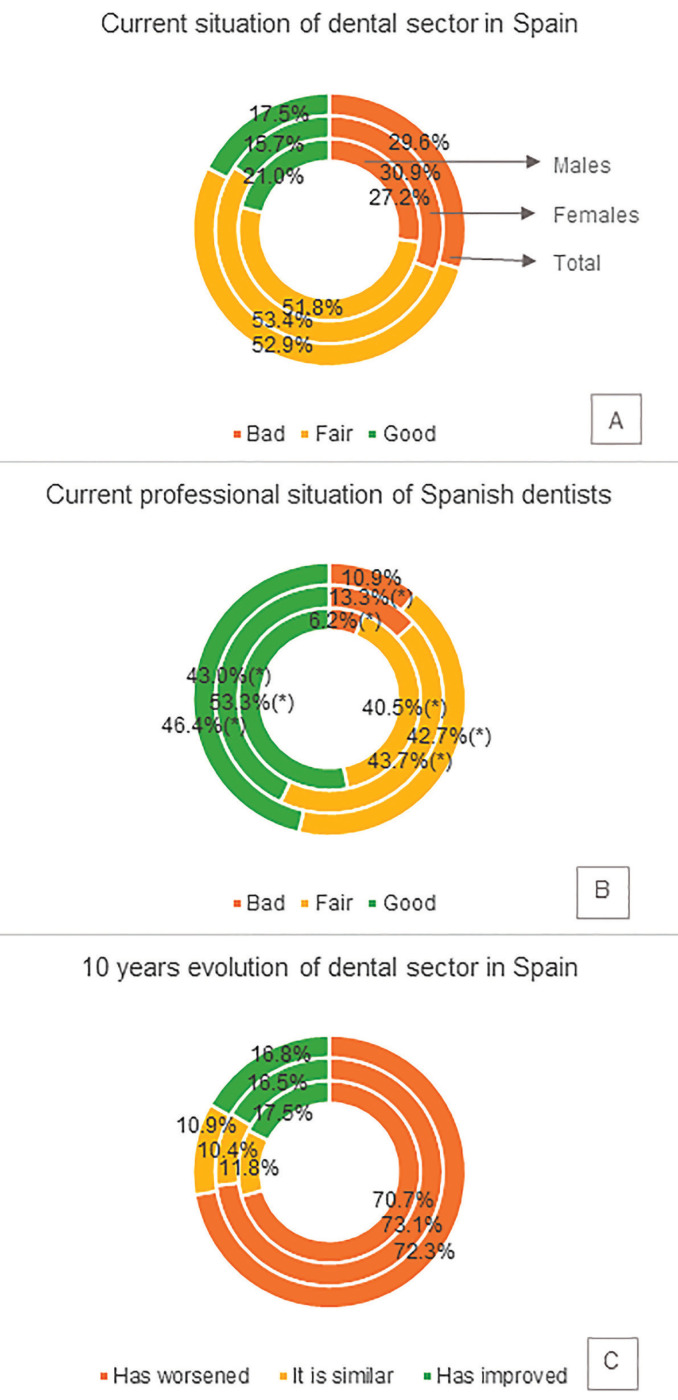
Average of valid responses to questions B1 (A),B3 (B) and B2 (C) of the questionnaire ( Table 1) expressed as percentages (*) Marks statistical significative differences between groups.

Most dentists describe the situation of the profession in Spain as “fair” or “bad” and a 72.3% considered that it “has had worsened” in the last 10 years (Fig. 1A,C). However, most of them considered themself in a “good” situation as a dentist, observing differences statistically significant between genders. Among women, a 13.3% described her situation as “bad” while among men they were only a 6.2% (Fig. 1B).

In terms of gender equity in the profession, almost everyone said that improvements have been made (only 11.4% marked the option of “no improvements”), although while 42.0% of male dentists considered that gender equity in dentistry has improved “a lot”, only 12.2% of feminine dentists perceived it in the same way (*p*<0.001).

- Influential factors for professional life success.

More than 90% of the participants considered important empathy and seriousness in order to achieve professional success in dentistry. Differences between genders were found for the influence of childcare, 54.1% of female dentists considered that it could be an influential factor for success, while only 22.4% of male dentists did (*p*<0.05) (Table 3).

**Table 3:**
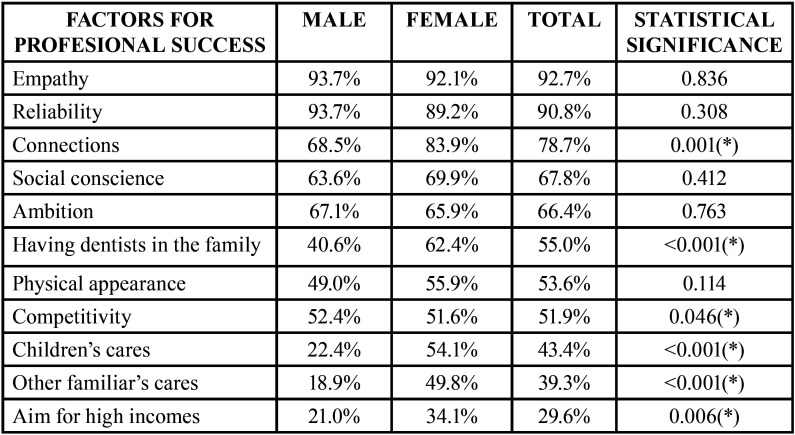
Average of affirmative responses expressed in percentages to the question C3: Do you think that the following aspects are important for achieving success in dentistry? (*) Marks statistical significative differences between genders.

Regarding the feminine presence in Spanish dentistry and their main organizations, three out of five people that answered, considered that the overall profession was equally represented by genders. However, only half of them (31.0%) considered that equality was shown in the General Council of Dentists (the main institution of Spanish dentists) and most of the female participants (49.5%) felt underrepresented (*p*<0.01).

- Equal opportunities and work-life balance in dentistry.

**Figure 2:**
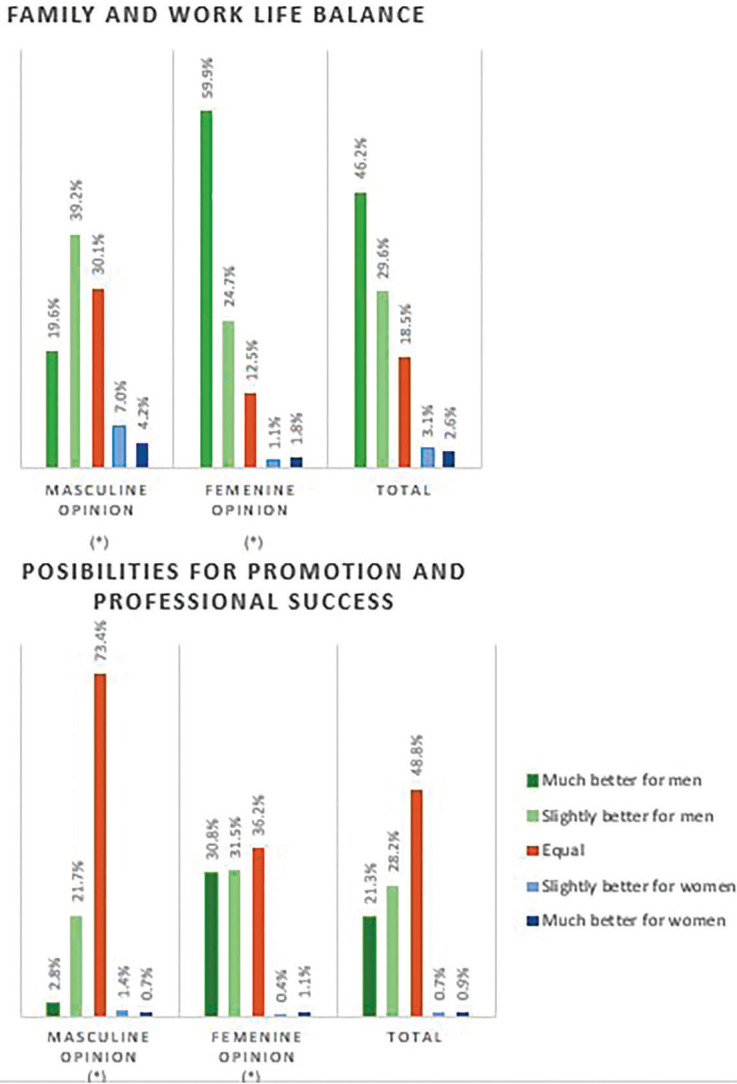
Average of affirmative responses for questions D1 (2) and D1 (4) of the questionnaire ( Table 1 cont.), expressed as percentages (*) Marks statistical significative differences between groups.

Dentists (75%) considered that men have more facilities to balance their family and work lives, existing significative differences of opinion between genders, male dentists believe more often that there are equal conditions for conciliating and for professional promotion (Fig. 2).

More than half of participants considered that men could have easier access to responsible positions in the General Council of Dentists (62.5%), in regional Professional Colleges (57.8%) and in scientific societies (58.3%). However, most people considered that in multidisciplinary dental clinics there was equal opportunity access to higher positions (56.9%).

Asked about the current conditions of work-life balance polices, most dentists described the maternity and paternity leave and flexible schedules as “insufficient or bad conditions”. However, among female dentists the percentages were higher (ranging from 54.5% to 73.4%) than the percentages of male dentists who considered the same (that ranged from 44.1 to 49.0%) (all p’s<0.001).

- Actions to promote gender equity in dentistry institutions.

Surveyed dentists showed different opinions based on their gender on whether if policies to guarantee gender equity should be a priority for main dental institutions in Spain. Most of the male dentists considered that these actions “shouldn’t be a priority” or “were not proceeding”, while for more than 70% of women, gender equity “should be a priority” for all institutions(*p*<0.01).

- Professional and socioeconomic situation of dentists.

**Figure 3:**
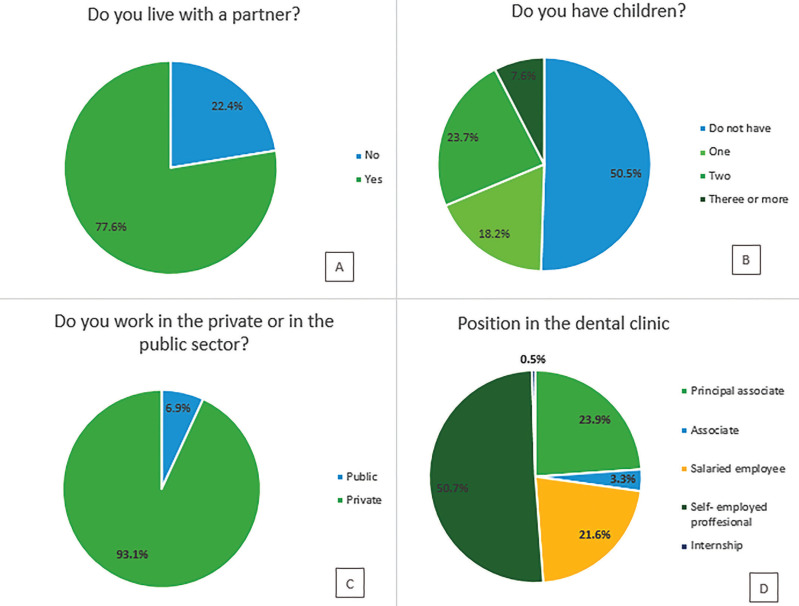
Personal and professional profile of surveyed dentists. Average of obtained responses for questions H1 (A), H2 (B), G3 (C) and G7 (D) of the questionnaire (Table 1 cont-2.) expressed as percentage.

Three out of four surveyed dentists stated living with a partner and half of them not having children (Fig. 3.A,B), despite 75% of the participants were aged between 25 and 55 years old (Table 2). Regarding the professional situation, only 6.9% worked for the public health system and more than 50% were self-employed professionals (Fig. 3.C,D). There weren’t gender differences for these questions, except for the dedication to different fields of dentistry. Male dentists dedicated to oral surgery were twice more than women, while paediatric dentists were women five times more than men (both *p*<0.001).

**Figure 4:**
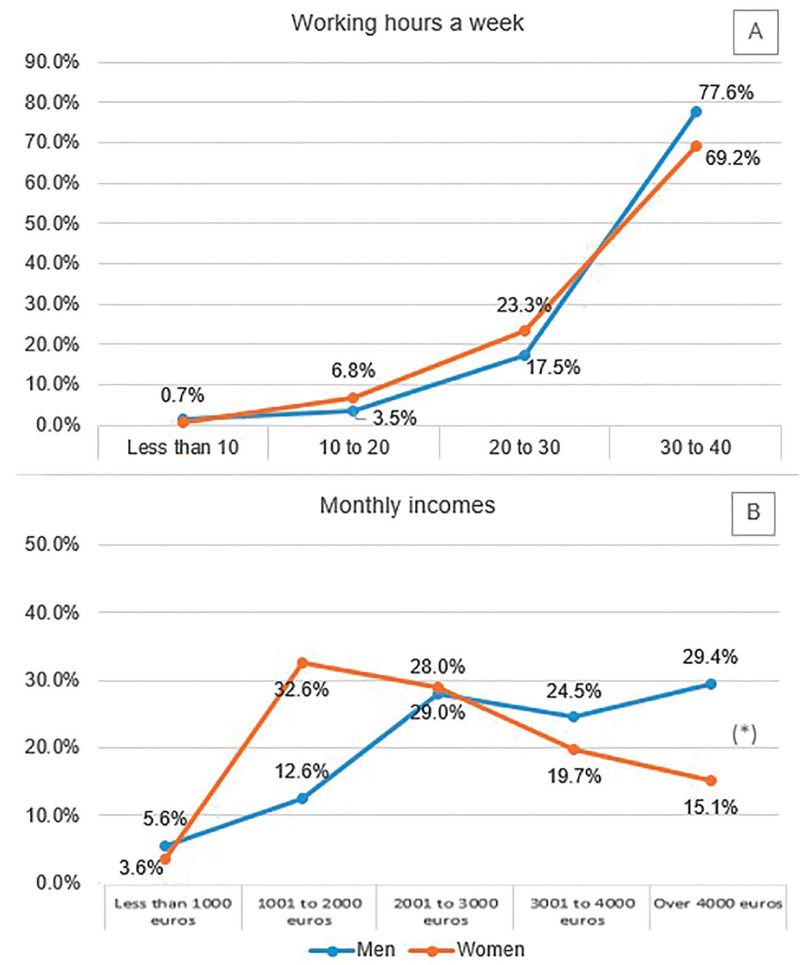
Graphic representation of percentual means for the obtained responses to questions G5 (A) and H4 (B) of the survey (Table 1 cont-2.). (*) Marks statistical significant differences between groups.

The participants were asked about their working hours per week and their professional income per month approximately. Regarding hours at work, despite the fact that the percentage of women that did not work full time was slightly higher, there were not statistically significant differences (*p*=0.19). In contrast, the differences were significant between genders for the monthly incomes (*p*<0.001). It was observed that there were more than twice as many female dentists than men who earned from 1001 to 2000 euros a month, while among those earning 4000 euros or more a month, male dentists almost doubled (29.4%) to feminine dentists (15.1%)(Fig. 4).

- Gender discrimination.

Participants were asked if they felt treated differently by their co-workers whether they were women or men. The majority of the male dentists felt treated equally by their female (96.5%) and male colleagues (95.8%). On the other hand, 26.5% of female dentists did not feel that their male colleagues treated them as equals (*p*<0.001).

Female dentists were asked if this unequal treatment was felt as discrimination or a negative trait. Most of them (51.6%) recognised that at least once in their career as dentists they have felt minus treated. This discrimination was felt repeatedly coming from patients for 38.4% of female dentists, having happened “sometimes” or “many times”.

They were also asked about what kind of discrimination they had suffered: more than 25% of surveyed dentists say they had felt underestimated because of their work or opinions, and more than 40% judged for their body or clothing “sometimes” or “many times”. Recurrent disrespectful comments and intimidations were expressed by around 20% of women and more than 10% (what would be equivalent to 2700 dentists approximately) acknowledged sexual harassment actions, at least once during their dental career.

## Discussion

For Spanish dentists, the situation of the profession is far from being satisfactory, having worsened over the last decade. However, they consider that the dental sector has improved in terms of gender equity, for example, achieving an equal number of male and female dentists. Therefore, as Pallavi and Rajkumar explained, this equity in representation could not have been translated as the end of all gender differences in the profession (20).

The results of this study show there are some aspects, in which gender equity is not perceived as a reality and these perceptions are coming to a greater extent from female participants. For example, only 30% of respondents considered that there was gender equity in the highest dental organization of the country (General Dental Council) and half of the female dentists considered their selves underrepresented in the institution.

On the other hand, socioeconomic data gathered in this survey, indicate that among dentists there could be economic gender inequities happening, best known as “the gender gap” (21). In the highest range of our salary scale of income, men were twice more than women, while they doubled male dentists among the ones earning from 1001 to 2000 euros a month.

These difficulties for female dentists to reach the leadership positions of the sector and the highest portion of incomes could be associated with the existence of the so-called “glass ceiling effect” in Spanish dentistry. The greater responsibilities in childcare and family life assumed by women are one of the previous explanations that have been given to this phenomenon (22,23). Female dentists could be giving more importance to the work-life balance, leading to less involvement in responsibility and leader jobs than men (20). Our results would follow this trend, since the surveyed women expressed higher levels of dissatisfaction with current conciliating policies and having children was considered more by them as an influential factor to achieve professional success.

The previous study conducted among Spanish lawyers by Camas García in 2017 (19) in which our questionnaire was based, found similar perceptions, what could explain that lawyers and dentists could be facing similar problems. Three out of four lawyers considered that not having children or other dependents was important to achieve professional success and 49% of the participants considered that women were underrepresented in the General Council of Spanish Lawyers.

No similar previous surveys carried among dental professionals were found, so we were unable to establish complete comparisons of results. However, the review by Binmadi and Alblowi in 2019 showed similar results on the prevalence of violent actions suffered by dentists. Most of them were coming from patients or their companions and were based on verbal accusations. Physic violence episodes were described from 4.6% to 22.0% of participants of the surveys studied (24), results that are in line with the prevalence found in the present study (12.6%). Further similar studies are needed to improve and compare the obtained results.

Moreover, it has been observed that there is a lack of data and socio-demographic information on dental workers, which needs to be urgently solved. Improving these data would allow us to understand better the current situation of the profession and reveal the problems that dentists may be facing. That may be worsening their work and personal life and, as a result, having consequences in the quality of the dental attention they provide to their patients (8,11,22,25). A better knowledge of these problems would also allow the regulatory organisms of the profession to induce faster, cheaper and more effective improvement strategies and policies.

In addition to the limited data available, our study has faced other limitations, as the sample. This study could not achieve a homogeneous geographical distribution throughout Spain, since not all professional College associations proceeded to the survey distribution among their registered dentists (the outbreak of Covid-19 pandemic in Spain complicated the distribution, due to some professional associations were only emailing urgent notifications). Although the response rate has been similar to the one obtained by Colin *et al*. in 2019 in other online questionnaire carried among dentists (26), the quantity of responses is lower than the one obtained by Bravo *et al*. in a previous survey of Spanish dentists (27). Likewise, only the complete questionnaires were analysed, reducing the evaluated responses in half, because most of the incomplete records did not comply with the socioeconomic questions (only 2.6% answered the first question in Section 8), which was important to achieve one of our objectives. This small sample and the voluntary nature of the survey, could have had caused overestimation of some results. Focusing on the social character of the sample, it can be observed that the percentage of women is slightly higher than registered dentists (66% versus 56%) and the age is similar to the average of the population of interest (55% of the sample has 40 years or less versus a 60% of the registered dentists) (13).

On the other hand, our study, due to it is observational and cross-sectional nature, can only determine possible associations without obtaining causal relationships, which would require further longitudinal studies. Furthermore, most of the variables studied are based on subjective opinions, which are difficult to quantify and extrapolate to the collective of dentists in Spain.

## Conclusions

1. Dentists expressed dissatisfaction with the current professional situation, considering that it had worsened in the last ten years. It is perceived that men have more facilities to access leadership positions in the dental sector and it is observed that they are more among dentists with better salaries.

2. In the personal life, they described their situation as good, although with deficiencies in reconciling work and family life. Among women, having children to attend was considered a factor that can affect their professional success and it was perceived that men could have an easier work-life balance. The persistence of some discriminatory behaviours towards female dentists was observed.

## References

[1] Boniol M, Mcisaac M, Xu L, Wuliji T, Diallo K, Campbell J (2019). Gender equity in the health workforce: Analysis of 104 countries. http://apps.who.int/bookorders.

[2] (2018). Profesionales Sanitarios Colegiados. Instituto Nacional de Estadística.

[3] George A (2007). Human resources for health: a gender analysis Background paper prepared for the Women and Gender Equity Knowledge Network and the Health Systems Knowledge Network of the WHO Commission on Social Determinants of Health Background to the Women and Gender Equity Knowledge Network. www.lshtm.ac.uk/hpu.

[4] (2006). Working together for health [Internet]. Vol. 9. World Health Organization.

[5] Langer A, Meleis A, Knaul FM, Atun R, Aran M, Arreola-Ornelas H (2015). The Lancet Commissions Women and Health: the key for sustainable development. Lancet.

[6] Buchan J, Dhillon IS, Campbell J (2017). Health Employment and Economic Growth: An Evidence Base. Health Employment and Economic Growth.

[7] (2017). A/RES/70/1. Transforming our world: the 2030 Agenda for Sustainable Development Transforming our world: the 2030 Agenda for Sustainable Development Preamble. United Nations Gen Assem Resolut. United Nations.

[8] (2016). Global strategy on human resources for health: Workforce 2030. World Health Organization.

[9] Connell R (2012). Gender, health and theory: Conceptualizing the issue, in local and world perspective. Soc Sci Med.

[10] Hammarström A, Johansson K, Annandale E, Ahlgren C, Aléx L, Christianson M (2014). Central gender theoretical concepts in health research: The state of the art. J Epidemiol Community Health.

[11] (2016). National Health Workforce Accounts: A Handbook. World Health Organization.

[12] GHWN's Hub on Health Workforce Data and Evidence: A concept note Background and justification. World Health Organization.

[13] (2020). Profesión en Cifras. Consejo General de Dentistas.

[14] (2019). INEbase. Profesionales sanitarios colegiados 2018. Instituto Nacional de Estadística.

[15] Bernal E, Sandra D, Juan GA, Fernando O, Sánchez Martínez I, Ramón J (2018). Health Syst Transit. Spain Health system review.

[16] Llodra Calvo JC (2010). La Demografía de los Dentistas en España. Situación pasada, presente y futura. Análisis 1994-2020. Consejo de Dentistas. Organización Colegial de Dentistas de España.

[17] von Elm E, Altman DG, Egger M, Pocock SJ, Gøtzsche PC, Vandenbroucke JP (2007). The Strengthening the Reporting of Observational Studies in Epidemiology (STROBE) statement: guidelines for reporting observational studies. Lancet.

[18] Consejo General de Colegios de Dentistas de España. Guia Dentistas.

[19] Camas García F (2017). La igualdad de género en la Abogacía Española: la evaluación actual de las abogadas y los abogados. Abogacía Española Consejo General.

[20] Pallavi S, Rajkumar G (2011). Professional practice among woman dentist. J Int Soc Prev Community Dent [Internet].

[21] Anghel B, De España B, Conde-Ruiz JI (2019). Brechas Salariales de Género en España.

[22] Tiwari T, Randall CL, Cohen L, Holtzmann J, Webster-Cyriaque J, Ajiboye S (2019). Gender Inequalities in the Dental Workforce: Global Perspectives. Adv Dent Res.

[23] Weyer B (2007). Twenty years later: Explaining the persistence of the glass ceiling for women leaders. Women Manag Rev.

[24] Binmadi NO, Alblowi JA (2019). Prevalence and policy of occupational violence against oral healthcare workers: systematic review and meta-analysis. BMC Oral Health.

[25] Boniol M (2018). National Health Workforce Accounts (NHWA) implementation for improving health workforce data and evidence. http://healthworkforce.eu/wp-content/uploads/2019/02/Mathieu-Boniol-SEPEN-Budapest2018.pdf.

[26] Collin V, Toon M, O'selmo E, Reynolds L, Whitehead P (2019). A survey of stress, burnout and well-being in UK dentists. Br Dent J.

[27] Crisis económica y síndrome de burnout en dentistas privados en España. Burnout, crisis económica y dentistas en España. Revista del Ilustre Consejo General de Colegios de Odontólogos y Estomatólogos de España (RCOE).

